# Case of Rupture of the Uterus during Labour, and Previous to Delivery

**Published:** 1840-09-26

**Authors:** James Mitchell

**Affiliations:** Philadelphia, Member of Dr. Warrington’s Obstetric Class


*Case of Rupture of the Uterus during Labour, and previous to Delivery.* (Reported by JAMES MITCHELL, Jr., of Philadelphia, Member of Dr. WARRINGTON's Obstetric Class.)

Mary M'C——, æt. thirty-five, native of Ireland, considerably under medium size. Four times pregnant. First child, (male,) was removed by forceps by Dr. ———. Two subsequent labours were very tedious, but resulted in the birth of daughters. During her present pregnancy she complained much of pain in the right side, with occasional sense of suffocation. Her abdomen was very prominent,—uterine contractions commenced early in the morning of the 5th of September, 1840. I received a note from Dr. W., who had assigned the case to me, to attend her in labour, during the afternoon. At 5½ P. M., the membranes forming a large pouch in the vagina, and the os uteri appearing to be well dilated, Dr. W. advised me to rupture the membranes, thinking that although the head was still at the superior strait, and apparently well situated, the more efficient contractions of the uterus would advance it. Shortly after I found the membranes had ruptured, and the waters were flowing off, without, however, advancing the head, which I thought I could feel in the left occipito-acetabular position. At a subsequent examination I found the cord had prolapsed on the left side of the pelvis. This I carried up upon the point of my finger, and lodged above the linea ileo-pectinea. At the next contraction, however, it descended. I therefore requested the advice and assistance of my obstetric teacher, Dr. Warrington; and it is with his permission that I extract from his note-book the record of the case. "Finding a considerable loop of cord hanging loose in the vagina, I carried it up upon the point of my finger, (as Mr. Mitchell had already done,) and then introduced a piece of soft sponge, to retain it above the superior strait, where I found the vault of the cranium situated with the occiput towards the left acetabulum. At every renewed contraction, however, the cord would descend, until I was convinced the child would be in danger from an arrest of its circulation. As the cord was thus in constant danger from compression, I thought it proper to expedite the delivery to save the child's life. The choice of means for this purpose lay between the use of the forceps and version by the feet. Version by the feet would not lessen the difficulty, as the cord might still be compressed by the head after the delivery of the body; whereas, if the cord could be kept up until the head had fairly engaged, the circulation through the placenta might be permitted to go on till complete extension (as in the apparently similar case of M. C., No. 390 of the table.) The forceps were therefore decided upon, and were applied with facility. The head, however, could be made to advance only very slightly, under the traction effort, aided by the very powerful contractions of the uterus. It receded as soon as the pain subsided. As the cord continued to force down the sponge, I was apprehensive for the child, and requested Mr. Mitchell to state the condition of things to Professor Hodge, one of the Consulting Physicians of the Dispensary. A severe indisposition prevented him from going out, but he advised the perforation of the head while the forceps were still applied to it. Having determined never to do this for the first time myself, unless in the presence of a senior accoucheur, while it was my good fortune to practise in a city so well supplied, I requested an interview with Drs. Meigs or Huston. While making renewed traction efforts, after receiving the proposition from Dr. Hodge, the forceps slipped off the child's head. Another careful examination satisfied me that the occiput presented either in the first or fourth position; and though the scalp was so considerably suggillated as to obscure the diagnosis, my impression remained strong that it was the first. The uterine contractions were very powerful, but appeared to make little impression on the head; I reapplied the instruments, and, as on the former occasion, without pain to the patient. I had ascertained during my examination that pulsation had entirely ceased in the cord, and that I need not feel any further anxiety for the life of the child; but while making renewed effort to assist its passage through the pelvis of the mother, with a view to her escape from serious consequences, the blades again slipped off. I then desisted, and determined to await the arrival of one of the medical friends I had sent for. In half an hour after, as the patient seemed fatigued and thirsty, I had a draught of cold water administered to her. Instantly a severe contraction took place, just at the termination of which she complained of extreme colic-like pain over the whole abdomen, and especially in the epigastric region. I could not satisfy myself whether this extremely distressing sensation depended upon severe spasm of the stomach and bowels from the cold drink, or on rupture of the uterus at or near its fundus. The *labour pains*, however, did not return, and there was no external hæmorrhage. I passed one hand into the vagina and os uteri, while I applied the other over the abdomen, but could perceive no change in the situation of the fœtus. The pulse was very frequent, but it remained full and strong. I at once gave her spirits of peppermint, and about fifty drops of laudanum. In twenty minutes she was more easy; her pulse rather feebler; her surface and extremities warm. Professor Huston came in about 10 o'clock, and examined her abdomen, and remarked 'that although there was some angularity in the abdominal tumour, there was not sufficient to justify a positive diagnosis of rupture of the uterus; that possibly, some fibres of the walls might have yielded.' Upon passing the hand into the vagina, he found the head at the superior strait. Upon the whole view of the case, he concurred with us in the apparent propriety of taking blood enough from the arm to lessen the force of the circulation, of fomenting the abdomen and vulva with warm hop cataplasms, repeating the anodyne, and meeting again at a quarter before 8 next morning, it being now near 12 o'clock at night of 5th inst.

"Patient was extremely restless during the night; would not permit the use of the fomentations. At the time appointed for the consultation, her pulse was very rapid, respiration laboured, countenance sunken, and all prospect of further interference was abandoned. She died at half past one, P. M., of that day, about fifteen hours after these abnormal symptoms had taken place.

"*Autopsy at* 10 *A. M.*, *Ninth month 7th.*—Present, Professors Hodge and Huston, Drs. Bond, Hallowell, W. Poyntell Johnston, Pleasants, Gee, and Warrington: Obstetric pupils, J. Mitchell, Jr., H. Selden, and B. F. Henden. Dr. W. Poyntell Johnston made an elliptic incision around the sides of the abdominal tumour, through the rami of the pubes, which were cut away with a saw, and gave a very fair view of the parts. The flap thus made, was placed up over the thorax, when the fœtus was found laying upon its left side, with its right hand partially over its face; its head in the direction of the first position of the vertex, but now thrown forward so as to bring the sagital suture to rest upon the upper edge of the pubis. The cord was around the child's neck, and part of it doubled down into the vagina. Upon carefully lifting up the child and transferring it to a table, the fundus and body of the uterus could readily be recognized, laying upon the right side of the lumbar region, well contracted, while the neck appeared to be elongated and connected with the vagina. Through a rent in the left lateral and anterior portion of the cervix, the fœtus had escaped. The placenta was detached, and lying loosely upon the posterior part of the uncontracted neck. A large quantity of blood had been poured out into the sub-peritoneal cellular membrane, though none had escaped per vaginam, perhaps from the complete obstruction which the head had offered while engaged in the upper strait.

Unfortunately, no measurements were taken of the diameters of the pelvis, neither was the weight of the child ascertained.

The autopsy cleared our diagnosis of the rupture of the uterus, about which we had had, at the moment of its occurrence, some doubts, particularly as to its being more than a separation of some of the fibres, without involving the peritoneal coat. As neither of us could feel the child distinctly through the parietes of the abdomen, it verified our diagnosis of the position of the cephalic presentation, which, though subsequently obscured by the tumidity of the scalp, was very satisfactorily made out for the application of the forceps. The parallelism of the sagital suture with the right anterior portion of the brim of the pelvis at the time of the autopsy, existing only in consequence of the recession of the head from its engagement in the upper strait as soon as the pressure of the uterus was removed.

From the situation of the point of rupture and that of the fœtus, it is evident that, after the accident occurred, the fundus and body continuing slowly to contract, must have put the remaining portion of the neck so much upon the stretch as to carry the lacerated edge of the contracting mass over, first, the shoulders, body, hips, and breech of the child, and subsequently to be drawn down under the body where it was situated entirely out of sight until the fœtus was lifted from the cavity of the abdomen.

"*Remarks.*—Had I acted upon the proposition of Prof. Hodge, I might have saved the mother by opening the head of the child; but having resolved never to perform embryulcia for the first time without having by me, if possible, a senior accoucheur, I determined to wait the arrival of Drs. Meigs or Huston. I have, moreover, an insuperable aversion to the use of destructive instruments while the fœtus is alive. But, had I foreseen the event of such delay in this case, I ought to have disregarded both the circumstances which influenced me to wait on the present occasion."

Dr. Warrington has preserved the pathological specimen, to be kept in his obstetrical cabinet at his lecture-room in the institution.

